# Recognition and stabilization of geranylgeranylated human Rab5 by the GDP Dissociation Inhibitor (GDI)

**DOI:** 10.1080/21541248.2017.1371268

**Published:** 2017-10-25

**Authors:** Eileen Edler, Matthias Stein

**Affiliations:** Molecular Simulations and Design Group, Max Planck Institute for Dynamics of Complex Technical Systems, Magdeburg, Germany

**Keywords:** GDP dissociation inhibitor (GDI), Rab proteins, molecular dynamics simulation, post-translational modification, protein-membrane interactions, prenyl binding pocket, peripheral membrane protein, biophysical modelling, biophysics of small GTPases, endocytosis, membrane trafficking, prenylation of GTPases, small GTPases and their effector proteins

## Abstract

The small GTPase Rab5 is the key regulator of early endosomal fusion. It is post-translationally modified by covalent attachment of two geranylgeranyl (GG) chains to adjacent cysteine residues of the C-terminal hypervariable region (HVR). The GDP dissociation inhibitor (GDI) recognizes membrane-associated Rab5(GDP) and serves to release it into the cytoplasm where it is kept in a soluble state. A detailed new structural and dynamic model for human Rab5(GDP) recognition and binding with human GDI at the early endosome membrane and in its dissociated state is presented. In the cytoplasm, the GDI protein accommodates the GG chains in a transient hydrophobic binding pocket. In solution, two different binding modes of the isoprenoid chains inserted into the hydrophobic pocket of the Rab5(GDP):GDI complex can be identified. This equilibrium between the two states helps to stabilize the protein-protein complex in solution. Interprotein contacts between the Rab5 switch regions and characteristic patches of GDI residues from the Rab binding platform (RBP) and the C-terminus coordinating region (CCR) reveal insight on the formation of such a stable complex. GDI binding to membrane-anchored Rab5(GDP) is initially mediated by the solvent accessible switch regions of the Rab-specific RBP. Formation of the membrane-associated Rab5(GDP):GDI complex induces a GDI reorientation to establish additional interactions with the Rab5 HVR. These results allow to devise a detailed structural model for the process of extraction of GG-Rab5(GDP) by GDI from the membrane and the dissociation from targeting factors and effector proteins prior to GDI binding.

## Introduction

Rab proteins belong to the Ras superfamily of small GTPases^1^ and form the largest subfamily with 62 members in humans.^2^ Rab proteins regulate intracellular vesicle trafficking and the transport between endocytic and secretory pathways. They are specifically localized to distinct membrane compartments thereby establishing membrane identity.^3,4^ Here, the focus is on Rab5, an early endosome marker and involved in early events of endocytosis, endosome fusion and the regulation of phagocytic transport.^5^

**Figure 1.**
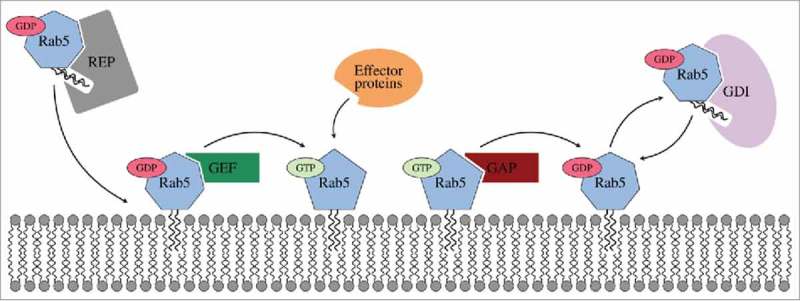
Inactive geranylgeranylated Rab5(GDP) is transferred to the membrane by Rab escort proteins (REPs) and subsequently activated by guanine nucleotide exchange factors (GEFs). Active Rab5 is exclusively membrane-associated and able to bind effector proteins. GTP hydrolysis is accelerated by GTPase activating proteins (GAPs). Inactive Rab5 is released from the membrane and maintained in the cytoplasm by a GDP dissociation inhibitor (GDI).

As small GTPases Rab proteins cycle between an inactive GDP-bound state (Rab(GDP)) and an active GTP-bound state (Rab(GTP)), only the latter being able to recruit signalling effector proteins (Fig. 1). Regulatory and effector protein recognition and binding is mediated via the nucleotide-state-specific flexible switch I and switch II regions. The nucleotide exchange cycle is also coupled to a transport process from the cytoplasm to the membrane.

Rab(GTP) is solely membrane-associated, while Rab(GDP) is found on the membrane in complex with guanine nucleotide exchange factors (GEFs) and in the cytosol in complex with the general Rab regulator GDP dissociation inhibitor (GDI) after deactivation by GTPase-activating proteins (GAPs).^6^ GDI serves to release Rab5(GDP) from membranes, to maintain Rab5 in the cytoplasm and to recycle it back to donor membranes. When forming the Rab(GDP):GDI protein-protein complex the hydrophobic lipid anchor is shielded from the solvent.^7^

In contrast to Rab escort proteins (REPs),^8^ GDIs selectively bind to post-translationally prenylated Rab(GDP) proteins in order to mobilise and recycle Rab(GDP).^9^ In the case of Rab5 proteins the posttranslational modification is a covalent attachment of two geranylgeranyl lipid moieties to two C-terminal cysteine residues Cys^212^ and Cys^213^. This lipid anchor is connected to the conserved, catalytic GTPase domain (G domain) via a long, disordered hypervariable region (HVR).^10^ These HVR are highly variable in sequence and initially thought to be responsible for specific Rab-membrane targeting^11,12^ which was later refuted.^13^

Valuable insight into mammalian and yeast Rab(GDP) and GDI interactions has already been obtained from protein crystallography. The crystal structures of free bovine alpha-isoform Rab GDI^14^ and in complex with a single geranylgeranyl cysteine amino acid^15^ have been determined. The positioning of the single prenyl chain was, however, above the GDI mobile effector loop (MEL, residues 215 to 221) and not in the prenyl binding pocket. The importance of including a full length lipidated GTPase to obtain a reliable GDI-protein model was later recognized.^16^ Later structures of the doubly prenylated yeast Ypt1:GDI complex^17^ as well as unprenylated yeast Ypt31:GDI and Sec4:GDI complexes^18^ provided valuable insights into the formation of protein-protein contacts. Structural data for human GDI plus the Rab5:GDI complex in either the soluble or membrane-bound form are not available. The bovine and yeast GDI is composed of two main units, a large multi-sheet domain I and an adjacent α-helical domain II. In yeast, three major protein-protein interactions sites have been identified. The Rab binding platform (RBP) in the GDI domain I interacts with conserved residues in the Rab switch regions. The C-terminus coordinating region (CCR) in the junction between both GDI domains establishes contacts with the Rab HVR. Third, there is a hydrophobic pocket within the GDI domain II which accommodates the prenyl groups of the Rab protein.^18^

Whereas membrane binding and orientation of human Rab5 in its GDP- and GTP-bound states^10^ and the geranylgeranyl lipid anchor-membrane interaction, diffusion and accumulation of phosphatidylinositol 3-phosphate (PI(3)P) lipids have been elucidated by molecular dynamics (MD) studies^19^ there is lack of information of full-length human Rab5 interactions with the GDI at the early endosome membrane and in the cytoplasm. We here describe the protein-protein interactions between geranylgeranylated membrane-bound Rab5(GDP) and GDI and the Rab5(GDP):GDI complex in the cytosol. Full-atomistic MD simulations have been shown to allow detailed qualitative insights into internal motions, the orientational flexibility of prenylated small GTPases at the membrane and into protein-protein interactions with high spatial resolution.^10^^,^^20^^,^^21^ With its key regulatory function in early endosomal trafficking, Rab5 is an important target for therapeutic interventions using structural^22^ and computational approaches.^23,24^

Here, we present a model for full-length human Rab5(GDP) in complex with human GDI. In the cytoplasm, Rab5(GDP):GDI protein-proteins interactions are classified along the GDI RBP, CCR and the prenyl binding pocket. We identified different GG-binding modes which resulted in different HVR-CCR interactions. The GG-binding pocket exists in an “open” or “close” conformation depending on the position of the Rab5 prenyl chains. Presence of the GG chains resulted in a transient pocket opening by a structural rearrangement of helix H1 in the GDI domain II. In the “close” conformation the GG chain cannot insert into the binding pocket which is shielded from the solvent. For the membrane-bound Rab:GDI complex, a first recognition of the nucleotide state of the GTPase by binding to the switch regions is established by the GDI RBP. In a second step, additional Rab-specific interactions are established re-orienting the GDI to allow further short range contacts to the HVR of Rab5. This leads to an opening of the prenyl binding pocket absent of direct contacts with the GG anchor. This shows that consecutive steps of Rab5(GDP):GDI complex recognition are required to finally extract the prenyl chains from the bilayer and stabilize the complex in the cytoplasm.

## Results

### A model for human GDI and positioning of the geranylgeranyl chains

**Figure 2.**
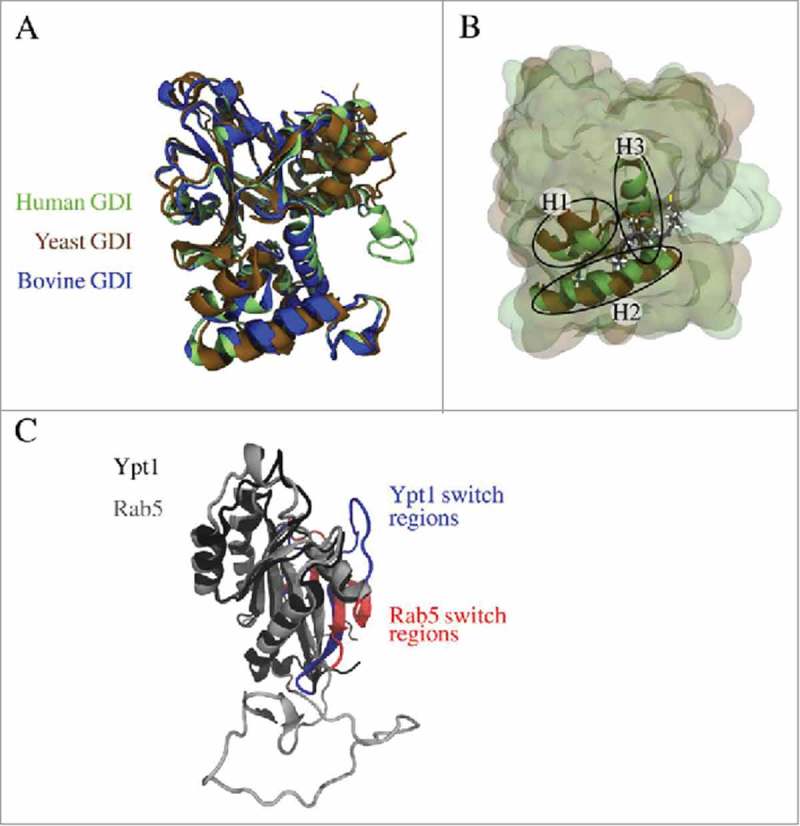
(A) Superposition of modelled human GDI (green) and X-ray structures of yeast GDI (PDB entry 2BCG,^17^ brown) and bovine GDI (PDB entry 1LV0,^15^ blue). (B) The geranylgeranyl binding pocket is formed by three helices (H1, H2, and H3) and accommodates the prenyl moieties of the small GTPase (from yeast 2BCG in brown, the modelled geranylgeranyl chains are coloured according to the atom types). (C) Superposition of Rab5 (grey) and Ypt1 (black) revealed structural differences in the GTPase switch I region coloured in red (Rab5) and blue (Ypt1), respectively. This is due to the fact that for Rab5(GDP) in complex with Rabaptin5 a conformation with an unusual β-strand in the switch I region was crystallized (PDB entry 1TU4^22^).

A structural model for human GDI (hGDI) with a root mean square deviation (RMSD) of only 0.015 nm to the bovine GDI (bGDI)^15^ and 0.187 nm from yeast (yGDI)^17^ was generated (Fig. 2A). The most prominent structural deviations between hGDI and yGDI are in a small inter-helical loop region (residues Phe^56^ to Arg^70^ in hGDI) encompassing the geranylgeranyl (GG) chain-binding pocket (GG-binding pocket), especially for helix 1 (H1, residues Thr^122^ to Ser^129^ in hGDI), as well as at the C-terminus. This originates from the different binding modes of the prenyl chains in the crystal structures of bGDI and yGDI (Fig. 2B). bGDI was crystallized with only a single GG ligand covalently attached to a sole cysteine amino acid. The binding site of this short chain was incorrectly assigned to be the prenyl binding pocket within the GDI domain I, below the Rab binding platform (RBP) and close to the mobile effector loop (MEL, hGDI residues 215 to 221).^15^ X-ray diffraction studies of the doubly prenylated yeast analogue Ypt1 in complex with yGDI later identified a different anchor-binding site within the GDI domain II and formed by helices H1, H2 and H3.^17,25^ Binding of the two prenyl chains resulted in a structural rearrangement of H1 disclosing a hydrophobic cavity (Fig. 2B). Consequently, the helices H1, H2, and H3 in domain II of hGDI derived from bGDI did not show the “open” conformation as in yGDI. The position of the Rab5 GG chains was therefore chosen according to the prenyl chain position in the Ypt1:GDI complex.

The Rab5(GDP):GDI protein-protein complex interface was modelled using the yeast Ypt1:GDI complex as a template. Superposition of both complexes is shown in Fig. 2C. The initial RMSD between human Rab5 and yeast G domains (Rab5: 16 to 184) was 0.479 nm. Structural differences in the switch I region of Rab5 (residues 44 to 66) result from crystallized conformations of Rab5(GDP) in complex with Rabaptin5. There are two conformations of GDP-bound small GTPases which differ in their switch I region. Complexed Rab5 displays additional β-strand formed by the switch I region^22^ whereas Ypt1 in complex with GDI exhibits a disordered switch I region. The hypervariable region HVR at the GTPase C-terminus (Rab5: 186 to 215) is highly variable in length for different Rab proteins. Visual inspection of the monoprenylated Ypt1:GDI complex reveals a very weak interaction between the Ypt1 hypervariable domain and yeast GDI. This helps to explain differences between Ypt1 and hRab5 HVR structures.

**Figure 3.**
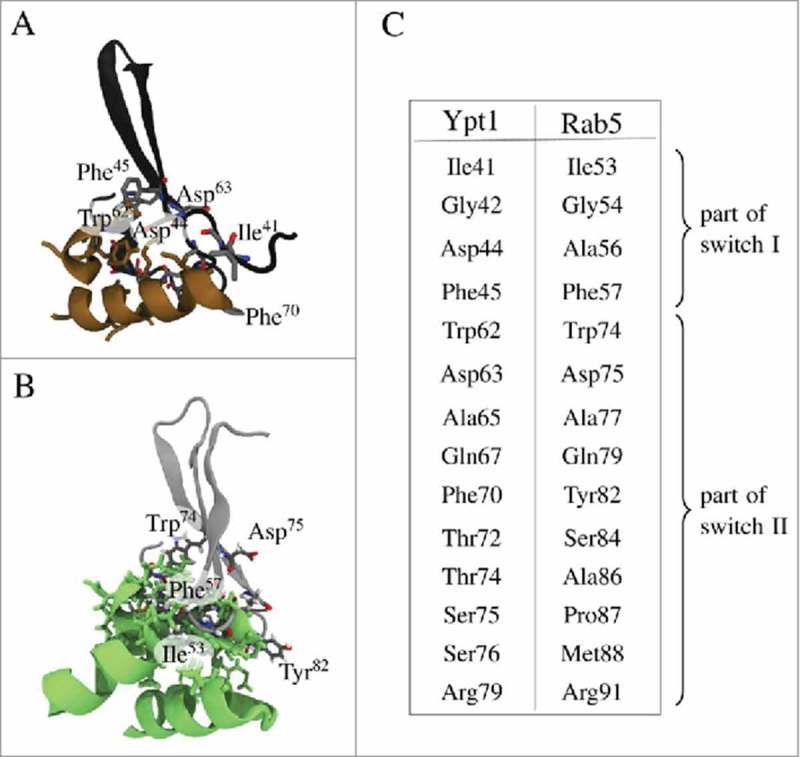
Details of interactions of the GDI Rab binding platform (RBP) in GDI domain I with the Rab binding epitope from switch I and switch II residues. (A) Interactions between yeast RBP (brown) and the Rab binding epitope from Ypt1 (black). (B) RBP in green (hGDI) interacting with hRab5 (grey). (C) Conserved residues in switch regions I and II which encompass the Rab binding epitope in yeast and human.

The Rab GDI binding epitope is highly conserved between yeast and human GTPase. In Ypt1 four conserved residues from switch I and ten residues from switch II constitute the Rab GDI binding epitope which interacts with the GDI Rab binding platform (RBP)^18^ (Fig. 3). In human Rab5 these residues have very similar physical properties. There are only a few minor non-conserving differences in the switch II region, namely non-polar Phe^70^ in Ypt1 is replaced by polar Tyr^82^ in Rab5, and Ypt1 residues Thr^74^, Ser^75^, and Ser^76^ are replaced by Ala^86^, Pro^87^, and Met^88^ in Rab5. Due to the ordered Rab5 switch I region in the initial model there were more contacts formed within the RBP of human Rab5(GDP):GDI complex (cf. Fig. 3A, B) which were later removed by refinement of the complex structure. Long MD simulations were performed in order to identify relevant interactions which stabilize the human Rab5(GDP):GDI protein-protein complex in the cytoplasm.

### The cytoplasmic Rab5(GDP):GDI complex

**Figure 4.**
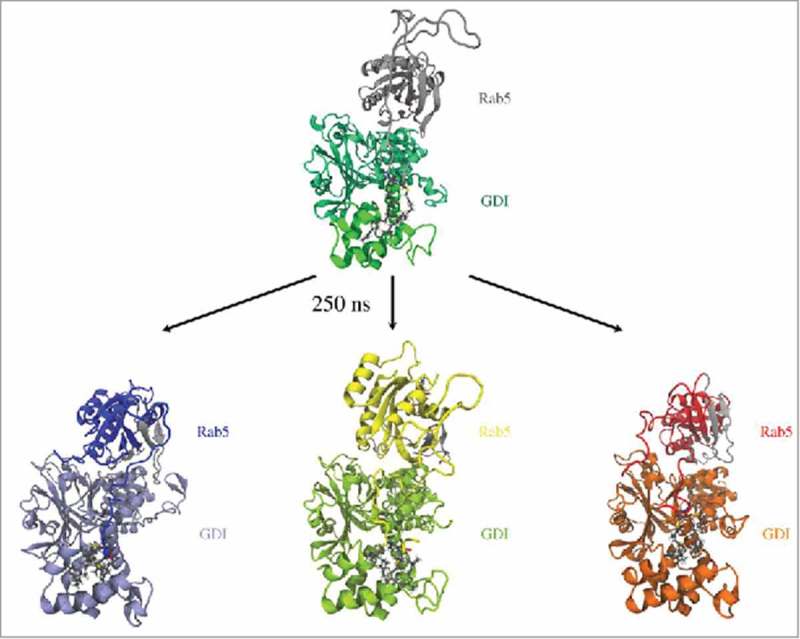
Initial (top) and snapshots from refined human Rab5(GDP):GDI complexes after 250 ns of MD simulation (bottom). Rab5 switch regions are coloured in grey.

The initial model of the Rab5(GDP):GDI complex in the cytoplasm was refined by three independent 250 ns production time full-atomistic MD simulations. All three runs started from an identical initial configuration (Fig. 4), but used different random velocity distributions. Three distinct binding modes of the GG-Rab5(GDP) to the GDI were observed. The finally obtained Rab5(GDP):GDI complex structures are shown in Fig. 4. Differences were found in the orientation of Rab5 relative to GDI and the Rab5 GG chain orientation in the binding pocket.

### Characterizing Rab(GDP):GDI protein-protein complexes in the cytoplasm

**Figure 5.**
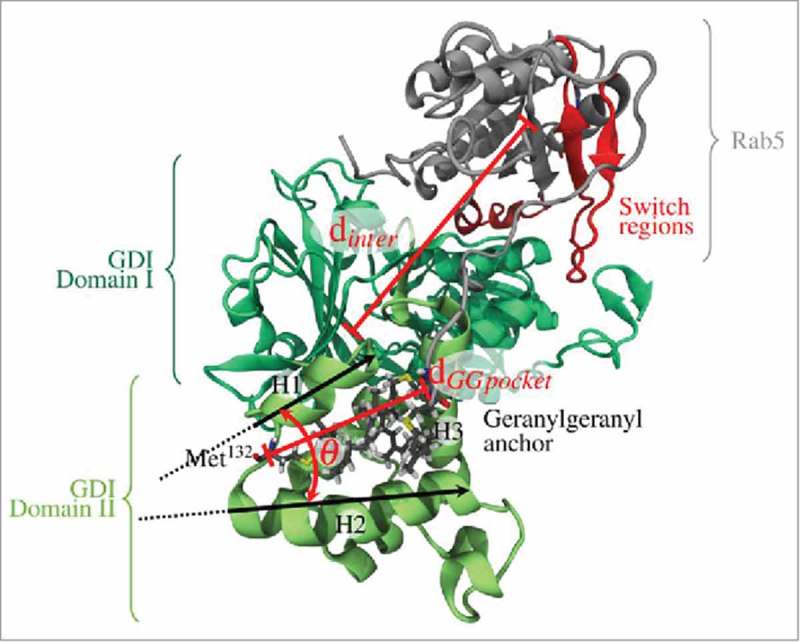
Definition of geometrical parameters to characterize different Rab5(GDP):GDI complex interaction modes: (1) the intermolecular distance between the Rab5 (grey) and GDI (green) centres of mass, d_inter_, (2) the angle between helices H1 and H2 of the GG-binding pocket, θ, as well as (3) the distance d_GGpocket_ between the GG cysteine Cα atoms and GDI Met^132^ at the bottom of the binding pocket.

The relative orientations of the binding partners in the Rab5(GDP):GDI complex were characterized by a set of structural parameters to discriminate between different interaction modes (Fig. 5). First, the intermolecular distance between the Rab5 G domain and GDI centres of mass, d_inter_, served as an indicator of the degree of protein-protein association. Two further geometrical parameters were defined to characterize the hydrophobic cavity: the angle θ between helices H1 and H2 serves as a measure for the degree of GG-binding pocket opening. The distance d_GGpocket_ between the completely buried Met^132^ residue and the GG-modified cysteine Cα atoms of cysteines 212 and 213, respectively, is a measure for the GG-chain insertion depth into the binding pocket (see Fig. 5).

### Interactions between the GDI Rab binding platform (RBP) and the Rab5 switch regions

The Rab binding platform (RBP) of the GDI is the major site of interaction with Rab proteins. It is localized at the top of domain I of the GDI (see Figure 5) and makes extensive contacts with the switch regions of Rab. This is to be expected since it distinguishes between active and inactive states of Rabs. The switch regions are highly conserved in the Rab protein family and also GDI residues that recognize those are conserved.

The RMSD and root mean square fluctuations (RMSF) of both Rab5 and GDI proteins are provided in the Supplementary Material (Fig. S2). Rab5 shows significant flexibility near the N- and C-terminal regions which was already observed before^10^ and explains their difficulty to crystallize. Moreover, Rab5 shows a degree of flexibility for the switch I (residues 44–66) and switch II (residues 75–91) regions (Fig. S2C). These Rab5 switch regions encompass the Rab5 binding epitope which forms contacts with the GDI RBP. The RBP comprises residues conserved between REPs (Rab escort protein) and GDI^14^^,^^26^^,^^27^ (hGDI residues Gly^232^ to Ile^259^).

**Figure 6.**
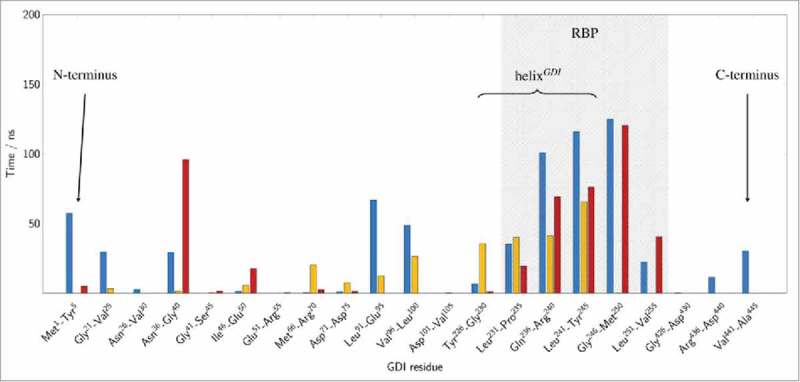
Structural mapping of cytoplasmic Rab5:GDI interactions. Average residence time of the GDI RBP residues in a 3 Å cutoff radius of the Rab5 binding epitope during final 200 ns (cytRun1: blue, cytRun2: yellow, cytRun3: red).

**Figure 7.**
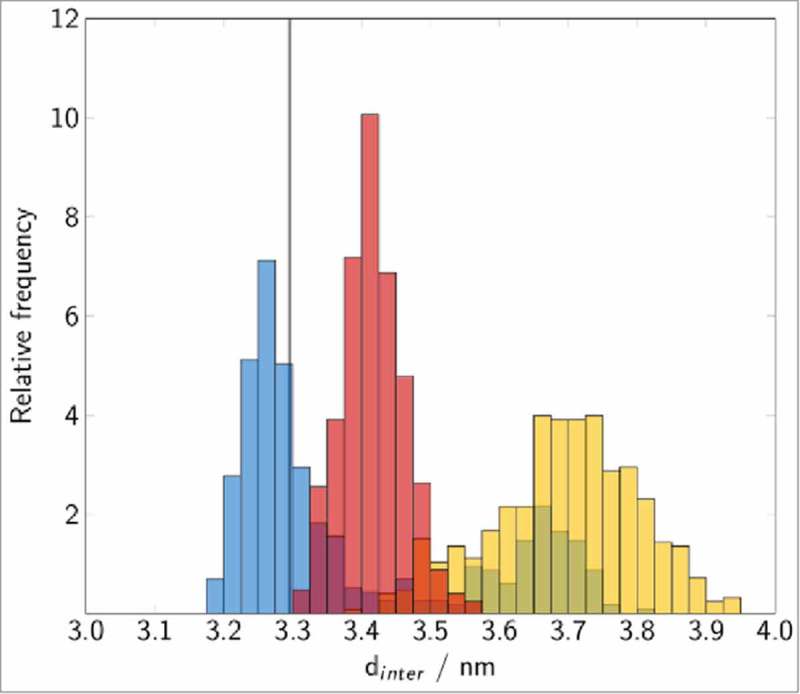
Distribution of the Rab5(GDP):GDI intermolecular distance in the cytoplasmic complex. The vertical line corresponds to the experimental value for Ypt1:yGDI.17 Histograms are coloured in blue (cytRun1), yellow (cytRun2) and red (cytRun3).

Two pre-dominant states of Rab5(GDP):GDI complexation could be distinguished (Fig. 6 and 7) according to the intermolecular distance and relative orientation of the Rab5 switch regions to the GDI RBP.

There is a ‘tightly’ bound complex which is the most frequent protein-protein complex from trajectories _cyt_Run1 and _cyt_Run3. This state is characterized by small Rab5:GDI intermolecular distances d_inter_ with centre-to-centre distances between 3.2 nm to 3.5 nm which is in good agreement with the Ypt1:yGDI intermolecular distance in 2BCG^17^ (blue and red bars in Fig. 7) and numerous interactions between the Rab5 binding epitope and the GDI RBP.

Four conserved residues in the switch I region, namely Ile^53^, Gly^54^, Ala^56^, and Phe^57^ (part of the Rab binding epitope in the yeast homolog Ypt1) are rather flexible in human Rab5 (Fig. S 2C). These residues are mainly located in the loop region connecting the two β-strands of switch I which explains the different switch I conformations when crystallized (see above). They primarily interact with residues from the GDI N- and C-termini in the tightly bound state as well as with the C-terminal part of the GDI RBP helix (helix^GDI^, GDI residues 230 to 246) (Fig. 6 blue and red bars).

In a second, ‘loosely’ bound state in _cyt_Run2 (Fig. 6 yellow bar), the intermolecular distance d_inter_ is larger with an average of 3.7 nm (yellow histogram in Fig. 7). Interactions of Rab5 switch I residues are limited to helix^GDI^ and absent of contacts with the GDI terminal regions. Consequently, the RMSF profile shows largest fluctuations for the switch I region in the this state (Fig. S 2C).

Ten residues of the Rab5 switch II region establish strong and frequent contacts with the GDI RBP. In the tightly bound state these contacts were made with a GDI loop region (residues 37 to 39), the entire helix^GDI^ as well as an associated extended region (GDI residues 247 to 251) (Fig. 6, blue and red bars). In contrast, in the loosely bound state the switch II region of Rab5 interact with helix^GDI^ and a short loop region between GDI residues 68 and 73 (Fig. 6, yellow bars).

We can thus define the RBP of hGDI according to major and frequent protein contacts with hRab5: from Figure 6 it becomes clear that the RBP (shaded grey area) reaches from residues 226 to 255 to bind the switch regions of hRab5.

### The GG-binding pocket and incorporation of Rab5 prenyl chains

The Rab(GDP):GDI complex in the cytosol accommodates the two long chain lipid geranylgeranyl anchors in a hydrophobic GG binding pocket formed by helices H1, H2 and H3 of GDI domain II. Several hydrophobic leucine residues (Leu^127^, Leu^131^, Leu^144^, Leu^189^, and Leu^216^) as well as Phe^140^ and Val^147^ make up the cavity formed by the three helices with residue Met^132^ being the most buried at the bottom of the GG-binding pocket. The positioning of the GG chains within the GDI domain II GG-binding pocket and the relative GG anchor orientation were monitored.

According to the experimental doubly prenylated Ypt1:GDI complex structure,^17^ in the initial configuration one GG chain was occupying the narrow hydrophobic cavity while the second chain was close to the GDI protein surface. During the MD simulations two significant GG anchoring positions were observed:

- Both GG chains fully inserted into the GG-binding pocket accompanied by a rearrangement of H1 and an opening of the cavity (_cyt_Run1).
- GG chains displaced from the hydrophobic binding pocket which remains in the “close” conformation (_cyt_Run2 and _cyt_Run3) (Fig. 4, lower panel and Supplementary Material Fig. S3).

Such a dynamic binding of GG-chains is in agreement with recent results from GG-HVR interactions within the hydrophobic interior of a phospholipid membrane. If one GG chain was deeply inserted into the bilayer, the second chain was bending towards the membrane surface.^19^ This high degree of structural and dynamic flexibility was shown to be similar to that of a truncated C-terminal Ras heptapeptide modified with two hexadecyl chains. The average insertion depth was found to be 38–39% of the bilayer thickness and showed that the geranylgeranyl anchor does not adopt a fully extended conformation and not fully penetrate the membrane.

**Figure 8.**
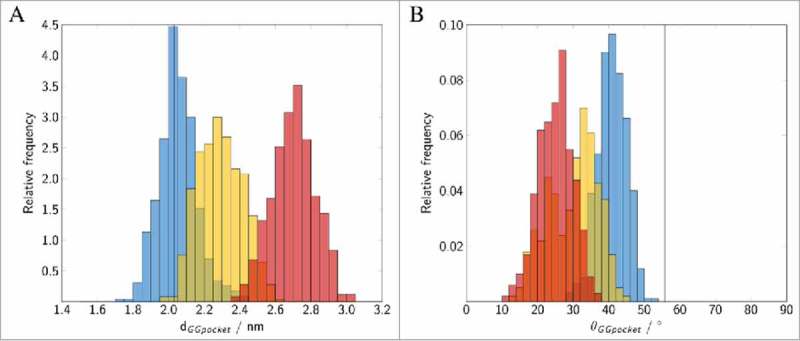
Binding of geranylgeranylated Rab5 to the GG-binding pocket of GDI. Distribution of (A) the distance between the GG chains and Met^132^, (B) the angle θ between helices H1 and H2 forming the GG-binding pocket. The vertical lines correspond to the GG-binding pocket distance and angle observed in the yeast complex from 2BCG.^17^ Data for the individual runs are coloured in blue (_cyt_Run1), yellow (_cyt_Run2) and red (_cyt_Run3).

In order to distinguish between the “open” and “close” conformations of the GG-binding pocket, the distance d_GGpocket_ between Met^132^ and the Cα atoms of Cys^212^ and Cys^213^ as well as the opening angle θ_GGpocket_ between H1 and H2 were monitored (Fig. 8).

**Figure 9.**
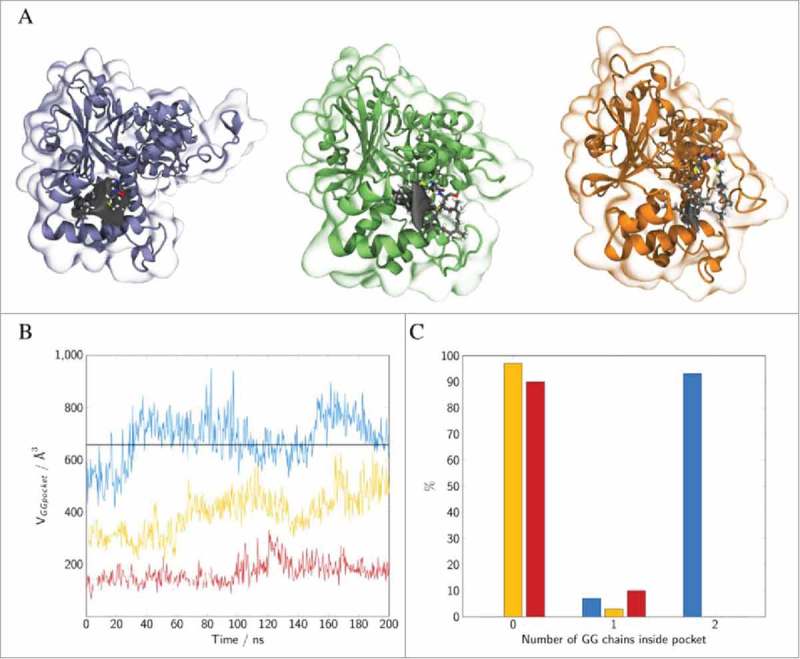
(A) Snapshots of GDI GG-binding pocket of Rab5:GDI complex for three independent runs (_cyt_Run1: left, _cyt_Run2: middle, _cyt_Run3: right). The GDI binding pocket persistent in 75% of all frames is displayed in grey. (B) The corresponding pocket volumes during the last 200 ns of MD simulation The black line refers to the pocket volume of 656.6 Å^3^ calculated for the GG-binding pocket in 2BCG.pdb. (C) Number of GG chains that are inserted within the GG-binding pocket. GG-chains are counted as “inside” the pocket for Met132 to head GG carbon atom distances <1.5 nm. Colours in diagrams as follows: _cyt_Run1: blue, _cyt_Run2: yellow, _cyt_Run3: red.

The open conformation is characterized by a small average Met-Cys distance of 2.0 nm and an opening angle θ>40°. This is in excellent agreement with results from the crystal structure of the yeast analogue complex which had a distance of 2.0 nm and an angle of 57°. In contrast, the close conformation shows a displacement of the GG chains from the pocket (large d_GGpocket_) and a decrease of angle θ (see Figure 8). The volume of the transient binding pocket can be used as a measure for the degree of opening of the binding pocket and the ability to accommodate the GG chains (see Fig. 9 and Fig. S3 in the SI). The time evolution of the volume of the GG-binding pockets in the trajectories was calculated using POVME 2.0^28,29^ (see Figure 9). Incorporation of two GG anchors in causes an outward movement of H1 (see above) and an opening of the hydrophobic cavity (see Figure 9). The average volume of the binding pocket of 692.1 Å^3^ (blue in Figure 9B) is very close to the 656.6 Å^3^ calculated for the GG-binding pocket in 2BCG.

In _cyt_Run2 and _cyt_Run3, the GG chains are at larger distance from the most buried Met^132^ residue and less deeply inserting into the GG-binding pocket. The GG chains remain close to the pocket entry of H1. The calculated pocket volumes were 396.4 Å^3^ for the ‘semi-open’ and 169.8 Å^3^ for the ‘close’ conformations of _cyt_Run2 and _cyt_Run3, respectively (Fig. 9). This is good agreement with the crystal structure of bovine GDI for which a volume of 108.6 Å^3^ was calculated. This allows an assignment of the ‘close’ pocket state to the bovine crystal structure.

The results are compared to binding pockets of various prenyl-binding proteins (Table 1). Mammalian RhoGDI *B. taurus* and *M. musculus* have volumes of 291.4 Å^3^ and 311.0 Å^3^, which is approximately half of the pocket volume in hRabGDI. Pocket volumes calculated for GGTase type-I and RabGGTase from rat range from 108.6 Å^3^ for GGTase type-I to 215.6 Å^3^ for RabGGTase. These pockets do, however, incorporate only one prenyl chain. The hydrophobic pockets of RabGGTase is occupied by a either a small geranylgeranyl pyrophosphate (GGPP) mimic or a truncated four residues peptide mimicking the Rab7 C-terminal region occupies the pocket which have smaller volumes between 206 and 216 Å^3^.

**Table 1.** Binding pocket volumes (in Å^3^) for prenyl-binding proteins. For human hGDI volumes are averaged over the final 200 ns each trajectory.

Protein	Volume / Å^3^	PDB entry	species
hRabGDI complexed with GG-Rab5, run 1	692.1 ± 79.4	-(present study)	*Homo sapiens*
hRabGDI complexed with GG-Rab5, run 2	396.4 ± 82.1	-(present study)	*Homo sapiens*
hRabGDI complexed with GG-Rab5, run 3	169.8 ± 42.0	-(present study)	*Homo sapiens*
yGDI complexed with doubly prenylated Ypt1l	656.6	2BCG^17^	*Saccharomyces cerevisiae*
yGDI complexed with Ypt31 (without prenyl)	648.6	3CPJ^18^	*Saccharomyces cerevisiae*
bRabGDI (without prenyl)	108.6	1LV0^15^	*Bos taurus*
bRhoGDI complexed with farnesylated RhoA	311.0	5FR2^50^	*Bos taurus*
RhoGDI complexed with GG-RhoA	291.4	4F38^51^	*Mus musculus*
Geranylgeranyltransferase (GGTase) type-I complexed with geranylgeranyl pyrophosphate (GGPP) and GG-peptide	146.4	1N4S^52^	*Rattus norvegicus*
RabGGTase complexed with GGPP	205.6	3DST^53^	*Rattus norvegicus*
RabGGTase complexed with Ser-Cys-Ser-Cys(GG) from Rab7	205.3	3DSV^53^	*Rattus norvegicus*
RabGGTase complexed with Ser-Cys(GG)-Ser-Cys from Rab7	215.6	3DSW^53^	*Rattus norvegicus*
RabGGTase complexed with Ser-Cys(GG)-Ser-Cys(GG) from Rab7	211.3	3DSX^53^	*Rattus norvegicus*

### Characterizing the Rab(GDP):GDI interface

**Figure 10.**
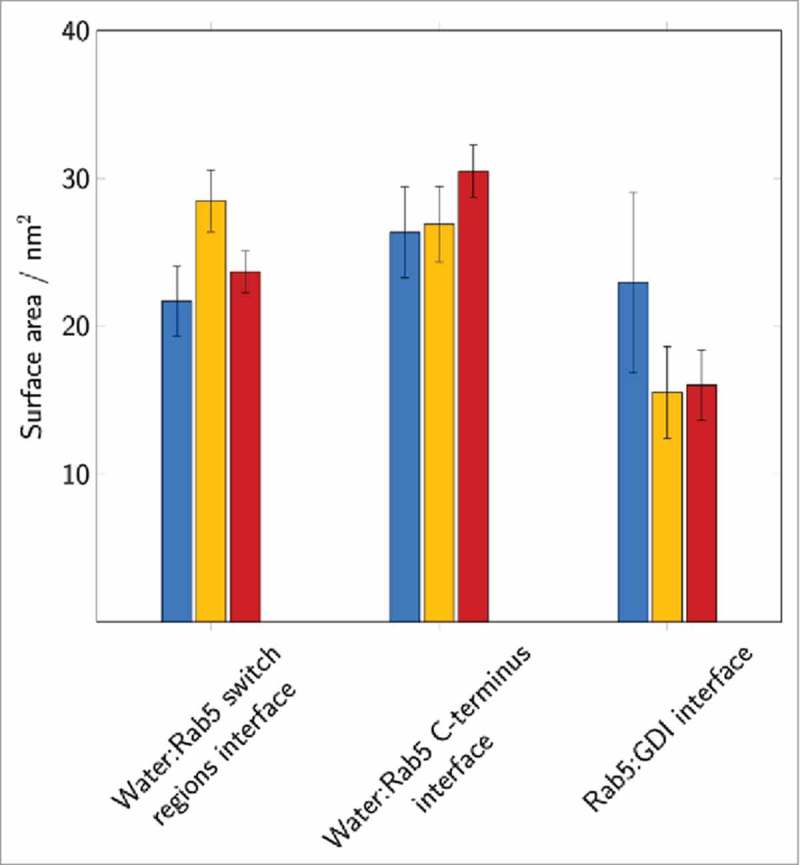
The solvent accessible surface area of the Rab5 switch regions and the C-terminal region as well as the total Rab5(GDP):GDI interface were averaged over 200 ns of MD simulation. Data for the individual runs are coloured in blue (_cyt_Run1), yellow (_cyt_Run2) and red (_cyt_Run3).

Calculation of the interface area of Rab5(GDP):GDI complexes allows a general classification of the protein-protein complex binding modes (Fig. 10). The protein-protein interface is largest in the tightly bound state (_cyt_Run1). The loosely bound states in _cyt_Run2 and _cyt_Run3 reveal a significantly smaller Rab5(GDP):GDI interface. The solvent accessible surface area (SASA) is used as a probe for the solvent exposure of parts of the Rab5 protein, e.g. the switch regions or the HVR, and in order to characterize the protein-protein interfaces of the Rab5(GDP):GDI complex (Fig. 10). The water exposition of the Rab5 switch regions was largest in conformations from _cyt_Run2. In contrast, in _cyt_Run3 a significant water exposure of the Rab5 C-terminal HVR was observed. This shows that the switch regions and the HVR of Rab proteins do contribute significantly to the protein-protein binding in loosely bound complexes. A large interprotein area is required for an insertion of GG chains and opening of the binding pocket.

### Interactions between the GDI C-terminus coordinating region (CCR) and the Rab5 hypervariable region (HVR)

**Figure 11.**
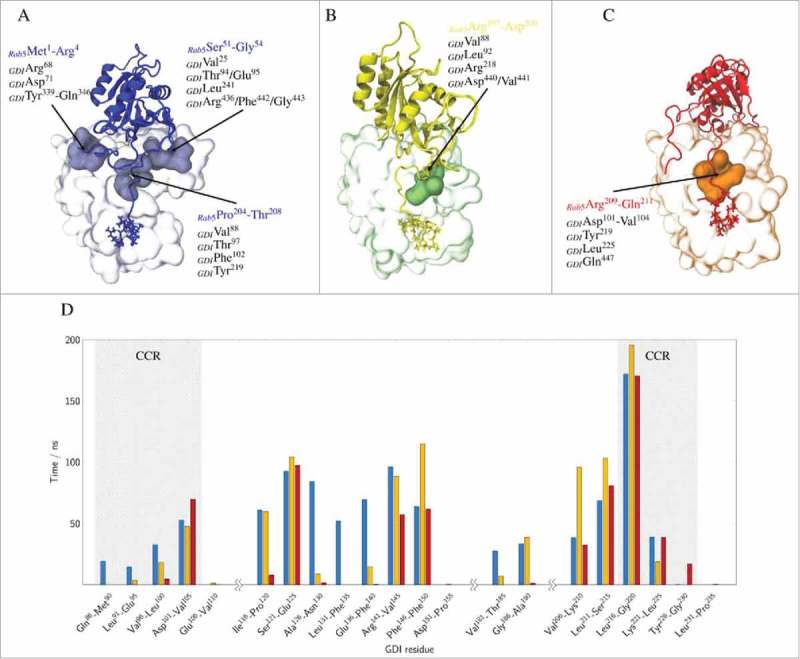
Top: GDI residues forming transient cavities that accommodate specific Rab5 regions are coloured in light blue, green or orange, respectively (A-C). Bottom: Average residence time of the GDI CCR residues that were found within a 3 Å cutoff of the Rab5 HVR (residues 190 to 215) are shown for the last 200 ns of the three independent runs (_cyt_Run1: blue, _cyt_Run2: yellow, _cyt_Run3: red) (D).

In addition to the RBP of domain I and the GG binding pocket of domain II, the C-terminal coordinating region of GDI located between the domains is a known interaction site for Rab proteins. The interactions of the HVR of Rab5 (residues 190–215) with the GDI CCR are shown in Fig. 11. The long Rab5 HVR forms interactions with hydrophobic GDI residues in all simulations (see Fig. 11A-C).

CCR residues Ile^100^, Thr^105^ and Tyr^227^ are known to affect binding and membrane extraction of Ypt1 by yeast GDI.^25,30^ They correspond to residues Leu^92^, Thr^97^ in domain I, and Tyr^219^ in domain II of human GDI. In the tightly bound state, the Rab5 switch regions interact strongly with the GDI RBP and the GG chains are deeply inserted into the GG-binding pocket, In addition, HVR residues 204 to 208 (PTQPT) preferentially form contacts with the GDI CCR cavity residues Val^88^, Thr^97^, and Phe^102^ from GDI domain I and Tyr^219^ and Leu^225^ from domain II. In _cyt_Run2 (Fig. 11B) the HVR solvent exposure is comparable to _cyt_Run1 (see above). HVR residues 197 to 200 (RGVD) also interact with the CCR, namely with residues Leu^92^, Thr^97^, Arg^98^ in domain I, Arg^218^ in domain II, and Asn^439^ at the GDI C-terminus but do so less frequently compared to the tightly bound form. In the complex with loosely bound GG chains from _cyt_Run3, the polar Rab5 HVR residues 209 to 211 (RNQ) preferentially interact with the GDI CCR residues Lys^103^ and Val^104^ in domain I, Arg^218^ and Tyr^219^ in domain II and Gln^447^ at the GDI C-terminus (Fig. 11C).

The simulations clearly reveal detailed polar or hydrophobic patches on the GDI domain I (residues Leu^92^ and Thr^97^) and domain II (residue Tyr^219^) interface to interact with the Rab5 HVR residues from Arg^197^ to Gln^211^. The exact residues interacting are depending on the positioning of the GG chains with respect to the binding pocket and the orientation of the flexible long HVR. This indicates that the long HVR of Rab5 is making a number of short range, very specific interactions with the CCR from GDI.

The HVR binding to the CCR functions like a Rab5-specific ‘zipper’ of van-der-Waals interactions. In the loosely bound state with a closed binding pocket, only few residues in vicinity of the Cys^212^ and Cys^213^ residues are binding to the GDI CCR. The largest part of the Rab5 HVR is not in contact with GDI but rather water-exposed and adopts a long and extended structure. In the ‘semi-open’ state, with GG chains partly inserted into the GG-binding pocket only residues at the N-terminal end of the HVR interact with the CCR. The Rab5 G-domain re-orients and makes few interactions between the switch regions and the GDI RBP. The tightly bound state may represent the cytoplasmic Rab5(GDP):GDI complex after Rab5 membrane extraction by GDI. It is characterized by a two-fold GG binding, an open binding pocket, a large protein-protein interface and a large number of specific HVR-CCR interactions. We hypothesize that the complexes with the GG chains partially inserted into the GG-binding pocket or close to the binding pocket represent intermediate states during the process of GG-Rab(GDP) extraction.

### MD simulations of membrane-bound Rab5(GDP):GDI complex

**Figure 12.**
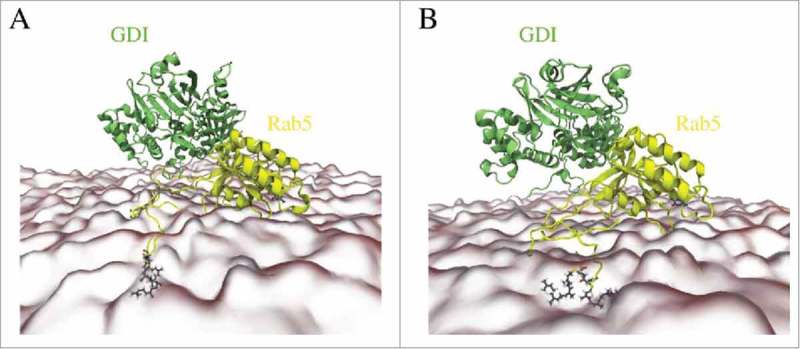
(A) Starting structure of membrane-bound human Rab5(GDP) and hGDI. (B) Refined Rab5(GDP):GDI complex after 250 ns MD simulation.

The GDI recognizes membrane-anchored GG-modified Rab5(GDP) to then extract it from the membrane and stabilize it in the cytoplasm. A 250 ns full-atomistic MD simulation with membrane-bound Rab5 obtained from previous studies^10^ comprising about 1,100.000 atoms was performed to investigate the interaction of GDI with membrane-associated Rab5. The cytosolic Rab:GDI complex was superimposed onto the membrane-bound Rab5(GDP) structure and without any steric clashes between GDI and membrane. The initial and final Rab5(GDP):GDI complex structures are shown in Fig. 12.

The RMSDs and RMSFs of membrane-bound Rab5(GDP):GDI in comparison to un-complexed membrane-bound Rab5(GDP) and cytoplasmic Rab5(GDP):GDI complex in the tightly bound state (_cyt_Run1) are provided in the Supplementary Material Fig.S4. The terminal regions of complexed Rab5 are stabilized by interactions with the membrane and GDI and display less flexibility compared to un-complexed Rab5 and cytoplasmic Rab5(GDP):GDI. In addition, membrane anchoring resulted in a significant stabilization of the Rab5 switch I region.

**Figure 13.**
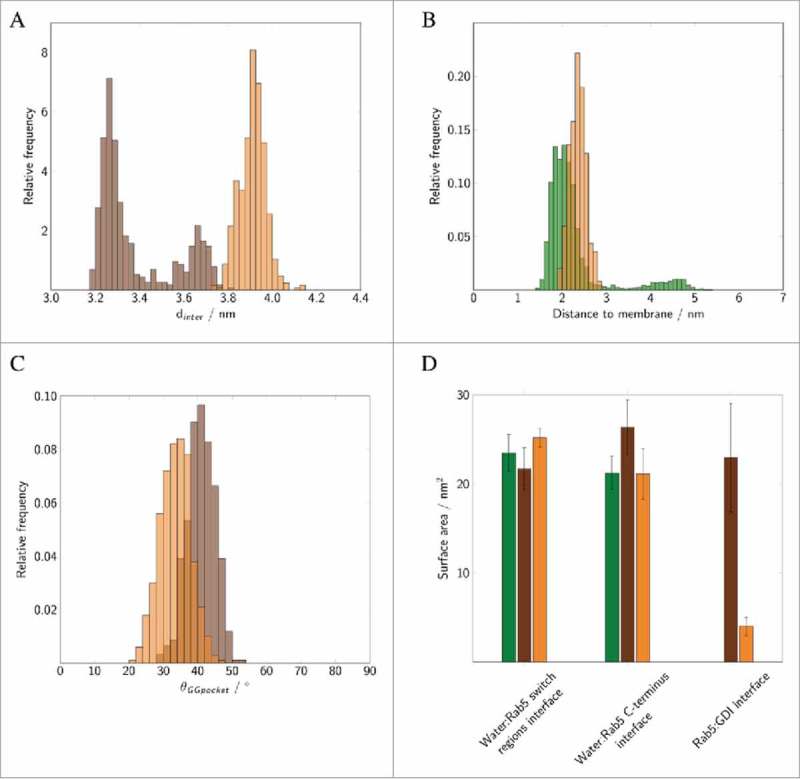
Distribution of (A) intermolecular distances of cytoplasmic (brown) and membrane-bound Rab5(GDP):GDI complex (orange). (B) Distances between the membrane and un-complexed Rab5(GDP) (green) and Rab5(GDP):GDI (orange). (C) Distribution of the opening angle θ of the GG-binding pocket in cytoplasmic (brown) and membrane-bound complex (orange). (D) The solvent accessible surface area (SASA) of the Rab5 switch regions and HVR as well as the total Rab5(GDP):GDI protein-protein interface area averaged over 200 ns of MD simulations. Data for un-complexed Rab5 are shown in green, for the cytoplasmic tightly bound Rab5(GDP):GDI complex in brown, and for the membrane-bound complex in orange. Data were averaged over three 500 ns MD simulations for un-complexed Rab5 from previous studies.

Structural parameters from _memb_Run are compared to un-complexed membrane-bound Rab5 and cytoplasmic Rab5(GDP):GDI (Fig. 13). The intermolecular distance d_inter_ between Rab5(GDP) and GDI is slightly larger in the membrane-bound complex compared to the cytoplasmic complex (Fig. 13A). The distances for the membrane-associated un-complexed Rab5(GDP) and complexed Rab5(GDP):GDI are comparable (∼ 2 – 2.5 Å). The average membrane-GDI distance remains at 4.4 nm during the simulation which indicates that the GDI remains bound to the Rab and does not form interactions with the membrane. Throughout the whole trajectory the Rab5(GDP):GDI complex remains membrane-associated and in an orientation with Rab5 tilted and close to the membrane surface and GDI in the cytosplasm, not interacting with the bilayer. The overall Rab5(GDP):GDI protein-protein interface is smaller for membrane-bound Rab5(GDP):GDI compare to the soluble complex due to initial interactions between the Rab switch regions and the RBP only (Fig. 13D). The Rab5 switch regions solvent accessibility is smallest for soluble Rab5(GDP):GDI which shows the tight protein-protein interaction in the cytosplasm and less so when membrane-bound. In the membrane-associated Rab(GDP) and when in complex with GDI, the C-terminus HVR is equally screened from the solvent. The HVR solvent accessibility is larger in the cytoplasmic complex. This indicates that both membrane surface and GDI binding have a similar effect on the HVR accessibility.

**Figure 14.**
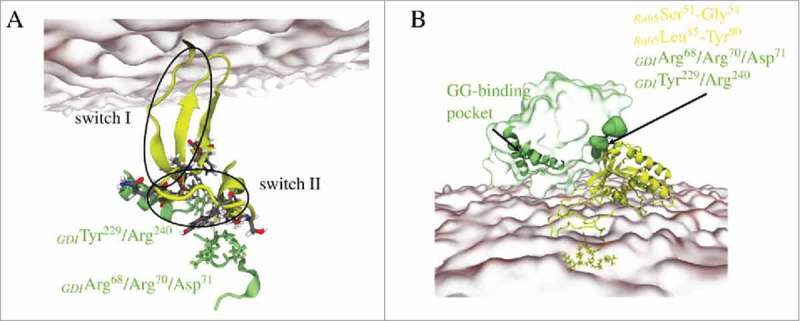
Refined structure of membrane-bound Rab5(GDP):GDI complex with highlighting (A) interactions between the Rab5 switch regions and the GDI RBP. (B) Cavities at the GDI surface accommodate Rab5 residues; helices H1, H2, and H3 form the GG-binding pocket. Rab5(GDP) and GDI are shown in yellow and green, respectively.

In the membrane-bound Rab5(GDP):GDI complex the opening angle θ of the GDI GG-binding pocket is slightly smaller compared to the ‘tight’ cytoplasmic complex with a deep insertion of the Rab5 prenyl chains (Fig. 13C). This is due to the absence of any GG chain from the binding pocket in the membrane-bound Rab5(GDP):GDI complex. The opening angle θ, however, is only slightly smaller than in the soluble complex which shows an additional opening induction of the hydrophobic binding pocket. Ignatev *et al.*^18^ suggested binding of the CCR to the HVR AXA box to induce such a structural change in H1. In our simulation of the membrane-bound complex such interactions could not be observed (Fig. 14).

Instead, interactions between the Rab5 switch regions and GDI domain I residues Arg^68^, Asp^71^, Tyr^229^, Arg^240^, Asn^342^, and Gln^346^ were identified (see Figure 14), which ensured a stable GDI-Rab complex formation even without binding of the Rab5 HVR or GG chains. These interactions may trigger such a pocket opening. The refinement of membrane-associated Rab(GDP):GDI led to a complex structure suitable to then form GDI-Rab5 HVR contacts and to subsequently transfer the GG chains from the membrane to the GG-binding pocket. Further work on longer simulation times beyond 250 ns is ongoing.

## Discussion

Molecular dynamics simulations of cytoplasmic and of membrane-bound Rab5(GDP):GDI complexes provide detailed insight into Rab5 recognition and stabilization by the GDI. Previous studies have focussed on binding of yeast Ypt1 and yeast GDI. After an intial structure of unspecific binding of a single anchoring molecule, the necessity of a full length lipidated Ypt1:GDI-protein complex was apparent.^16^ Structures of the soluble doubly prenylated yeast Ypt1:GDI complex^17^ and unprenylated yeast Ypt31:GDI and Sec4:GDI^18^ provided first insights into Rab-GDI binding but were limited to the cytoplasmic state. In the cytoplasm, the yeast analogue Ypt1 forms specific interactions with the HVR AXA box to induce structural changes in helix H1 which cannot be found for human Rab5. Protein X-ray crystallography cannot reveal information about the membrane-associated Rab(GDP) and its GDI binding. Thus, our work is the first to provide a structural model of human Rab5(GDP):GDI complex formation at the early endosome membrane and its stabilization in the cytoplasm by additional hydrophobic interactions. At the membrane, initial complexation of the Rab switch II region is mediated by Rab-specific interactions with the GDI RBP. In contrast, the switch I region in the Rab(GDP):GDI complex shows no significant difference in fluctuations between cytoplasmic and membrane-bound forms. This is consistent with the smaller contribution of Rab5 switch I to interactions with the GDI RBP and indicates a stronger involvement in interactions with the membrane. The Rab5 C-terminal HVR is a significant stabilized by forming interaction with the GDI CCR.

**Figure 15.**
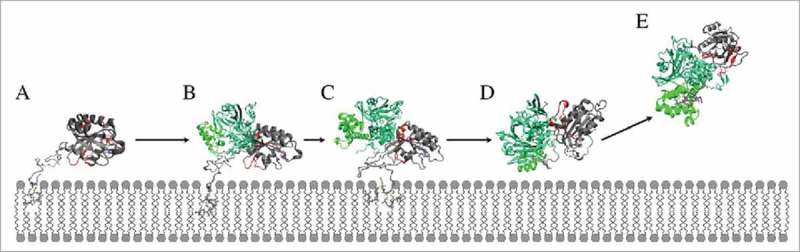
Mechanism of GG-Rab5 extraction from the endosome membrane into the cytoplasm. A sequential recognition and stabilization proceeds from (A) membrane-bound un-complexed Rab5(GDP) tilted towards the membrane surface with only partially accessible switch regions. (B) GDI recognizes Rab5 via its RBP and forms initial contacts with the switch regions. (C) The orientation of GDI slightly changes in order to allow interactions of the GDI CCR and the Rab5 HVR while the GG-binding pocket opens. (D) When the GG chains are accommodated within the GG-binding pocket, the Rab5(GDP):GDI complex detaches from the membrane. (E) The soluble complex is characterized by 1) manifold interactions between the GDI RBP and Rab5 switch regions, 2) Rab-specific recognition of Rab5 HVR by the GDI CCR, and 3) an opening of the GG-binding pocket with Rab5 GG-chains inserted.

The cytoplasmic simulations reveal two distinct interaction modes depending on the contacts formed between Rab5 and the three GDI interaction sites, namely the RBP, the CCR, and the GG-binding pocket.^18^ A ‘tightly’ bound state is characterized by a deep insertion of the GG chains into the GG-binding pocket and interactions of a central part of the Rab5 HVR (residues Pro^204^ to Thr^208^) with the CCR. This enables the Rab5 G domain to adopt an optimal orientation to interact with the switch regions with the GDI RBP. In contrast, the ‘loosely’ bound state exhibited a much smaller Rab5(GDP):GDI interface with no deeply inserted GG chains and significant less interprotein contacts being formed.Therefore, the loosely bound state may represent an intermediate state during the process of Rab5 membrane extraction by GDI (Fig. 15).

The flexibility and fluctuation of GG chain occupation of the binding pocket is in agreement with structural studies of the GG-HVR in the membrane^19^ and X-ray diffraction studies of the doubly prenylated yeast Ypt1:GDI complex. Only one GG chain was deeply inserted into the hydrophobic cavity whereas the other GG chain was bound in proximity to the binding pocket.^17^ This degree of fluctuations of doubly geranylgeranylated Rab binding is believed to stabilize the Rab:GDI complex in solution.

Incorporation of the GG chain is facilitated by an opening of the hydrophobic binding pocket. The concept of prenyl chain-induced structural rearrangements of a binding pocket is also observed for the Rho-associated counterpart of RabGDI.^31^ GG binding pockets with different pocket volumes are indicators of the degree of opening of the pocket. GGTases of type-I and the RabGGTase are known to incorporate only one GG group at a time and transfer GG chains in a consecutive one-chain-transfer reaction.^32^ They display significantly smaller binding pockets than the ‘tightly’ bound Rab(GDP):GDI complex in solution and comparable to the ‘loosely’ form. Since Rho GTPases are modified by either one farnesyl or one GG chain, this smaller pocket volume is sufficient to include the hydrophobic isoprenoid group. Large structural perturbations have been described to occur during insertion of the prenyl groups of Rho GTPases into the binding pocket of the RhoGDI. Steric hindrances are removed by displacing two β-strands and multiple associated amino acid residues. For the Rab5-GDI only minor structural rearrangements by helix H1 of the GG binding pocket are sufficient to enable GG binding.

Upon binding to the membrane-anchored Rab5(GDP) initial recognition of the switch regions by the GDI RBP is the first step of complex formation and sufficient for a stable complex formation at the membrane. The conformation and also their orientation at the membrane surface are characteristic for the GDP-bound inactive state. The Rab switch regions are conserved among the 60 human Rab proteins, and also GDI RBP patches are conserved. The GDI then first recognizes the inactive Rab(GDP) state and establishes mostly long range polar interactions between RBP and the Rab switch regions which encompass the Rab binding epitope. Following the initial recognition by the RBP, the GDI CCR re-orients to enable short range van-der-Waals contact formation with the Rab5 HVR which results in an opening of the GG-binding pocket. This re-orientation of the Rab(GDP):GDI complex at the membrane may prepare the membrane extraction process by reducing the HVR hydrophobic interaction with the membrane. Such a ‘twisting’ mode indicates that targeting factors and/or effector proteins must have dissociated from the Rab protein prior to GDI binding. During the re-orientation the GDI mobile effector loop (MEL) approaches the membrane surface, which is critical for GDI-membrane association.^33,34^

HVRs of human Rab proteins are highly divergent in sequence and were postulated to be responsible for Rab targeting to specific membranes or compartments.^11^^,^^12^^,^^35^^,^^36^ This would require the HVR to be solvent accessible. An exchange of the Rab HVR to those of other Rab proteins, however, did not affect the correct Rab targeting.^13^ This is in agreement with our study which shows that the HVR is undergoing an unspecific hydrophobic binding to the CCR of GDI and is solvent inaccessible in both the membrane and in the cytoplasmic complex. HVR residues binding to the CCR vary between the tightly and loosely bound states, thus supporting the idea that the HVR is a non-specific site of interaction with the GDI. This explains the presence of two human RabGDI isoforms to regulate the unspecific membrane extraction of a more than 50 different human Rab proteins.

HVR residues 204 to 208 (PTQPT) form contacts with the GDI CCR residues Val^88^, Thr^97^, and Phe^102^ from domain I and Tyr^219^ and Leu^225^ from domain II. These HVR residues, however, are not conserved among Rab5 isoforms (Rab5a, Rab5b, Rab5c) and neither among the large number of human Rab proteins (see Supplementary Material Fig.S5). The surface binding site CCR for the hypervariable domains allows GDI to interact with Rab hypervariable domains of different lengths. Our simulations indicate it is that rather the length of the HVR, e.g. the number of amino acids between the prenylation motif and G-domain that is driving the orientation and the interaction of the Rab G-domain with the GDI RBP. This has also been shown in several studies with membrane-bound small GTPases.^10^^,^^20^^,^^21^^,^^37^ Thus, binding the HVR at residues closer to the C-terminus results in tight interactions between the Rab5 switch regions and the GDI RBP while at the same time maintains rotational and translational flexibility of the G-domain.

We now have structural information regarding Rab:GDI complex recognition and stabilization at the membrane and in the cytoplasm. Doubly geranylgeranylated HVRs extraction from a negatively charged early endosome membrane causes only small perturbations within the membrane due to lipid reorganization during the extraction process. The free energy to fully extract a truncated HVR^206–215^ from such a charged membrane was calculated to be about 124 kJ/mol.^19^ It remains interesting to see how the GDI overcomes this free energy difference by incorporating the GG hydrophobic anchor into the hydrophobic binding pocket and thus reduces its energetically unfavourable solvent exposure.

## Methods

### Structural model generation

Based on the X-ray structure of GDIα from *B. taurus* (1LV0) at 1.8 Ǟ resolution^15^ a model for human GDI was created using MODELLER 9.12.^38^ Sequence alignment of human and bovine GDI revealed a sequence identity of >98% with only seven amino acids differing between both sequences. The top ranked Discrete Optimised Protein Energy (DOPE) score^39^ structure was further refined with MODELLER by remodelling the flexible loop region at the GDI C-terminus (residues 432 to 447). Based on the lowest DOPE score model this procedure led to 100 refined models which were clustered into five groups based on their root mean square deviations (RMSD). The structure closest to the average of the largest cluster was chosen as model for GDI.

For human Rab5(GDP), a full-length model of Rab5(GDP) post-translationally modified by two geranylgeranyl chains at residues Cys^212^ and Cys^213^ was taken from a previous studies.^10^ A ‘tilted orientation’ of membrane-bound Rab5(GDP) with a “buried switch” conformation was found. With its rather extended, solvent exposed HVR structure this model represented a suitable initial conformation for membrane-associated Rab5(GDP):GDI complex formation.

As a starting model for the Rab(GDP):GDI protein-protein complex and positioning of the geranylgeranyl chains the experimentally resolved Ypt1:GDI complex (PDB entry 2BCG)^17^ from *S. cerevisiae* was taken as a template. Superposition of human and yeast GDI showed a high conservation of secondary structure elements. The Rab5 geranylgeranyl chains were placed in accordance with the prenyl chains from the yeast complex. The human Rab5(GDP):GDI complex was first refined by energy minimization with constrained GG chain positions to remove unrealistic HVR orientations. A figure showing the Rab5(GDP):GDI complex and the exact location of the GG chains before and after minimization is provided in the Supplementary Material (Fig.S1, SI).

### Molecular dynamics simulations

Full-atomistic MD simulations were performed using NAMD2.9^40^ and the CHARMM36 force fields for proteins,^41^ lipids^42-44^ and nucleic acids.^45^ For the GDP nucleotide, nucleic acids parameters were combined with parameters for the phosphate group analogously to ADP.^46^ Force field parameters and topologies for the geranylgeranylated cysteine residue (hereafter called GG-Cys) were taken from previous studies.^19^ These were carefully evaluated and compared to quantum chemically calculated partial charges. The Rab5(GDP):GDI system was solvated in explicit TIP3P water^47^ and ionized to a salt concentration of 0.15 M sodium chloride. Prior to the MD simulations the system was energy minimized and gradually heated to 310 K. Subsequently, a 2 ns equilibration with fixed protein coordinates was performed. Three independent MD simulations of the cytoplasmic Rab5(GDP):GDI complex were performed (_cyt_Run1, _cyt_Run2, _cyt_Run3), each for 250 ns with the first 50 ns representing the equilibration time which was not considered in trajectory analyses. Therefore, the total production simulation time was 0.6 µs.

The membrane-bound Rab5(GDP):GDI complex starting configuration was generated from the cytosolic complex of _cyt_Run2 superimposed onto the membrane-anchored Rab5(GDP) from previous 500 ns full-atomistic MD simulations.^10^ Due to the huge system size of approximately 1,100.000 atoms, only one 250 ns trajectory of the membrane-associated Rab5(GDP):GDI complex was performed (_memb_Run).

The membrane composition corresponded to that of an early endosome (EE): palmitoyl-oleoyl-phosphatidylcholine (POPC, 17.8%), cholesterol (29.7%), palmitoyl-sphingomyelin (PSM, 9.9%), palmitoyl-oleoyl-phosphatidylethanolamine (POPE, 26.7%), palmitoyl-oleoyl-phosphatidylserine (POPS, 10.9%), and phosphatidylinositol 3-phosphate (PI(3)P, 5.0%). The production runs were performed in an NPT ensemble with constant particle number, pressure and temperature and periodic boundary conditions. Pressure was controlled using Langevin dynamics^48^ with an anisotropic pressure coupling in case of the cytoplasmic simulations. A semi-isotropic pressure coupling was applied for protein-membrane simulations. In combination with the SHAKE algorithm an integrator time step of 2 fs was used.

The SASA values for the individual amino acids “X” were calculated using the VMD “measure sasa” command and normalized using the experimentally derived values of the residue in a Gly-X-Gly tripeptide by Miller et al.^49^

## Supplementary Material

KSGT_A_1371268_supplemental.zip
