# Supplementary material for: Recognition and stabilization of geranylgeranylated human Rab5 by the GDP Dissociation Inhibitor (GDI)
Source: Small GTPases. 2017 Oct 25;10(3):227–42. doi: 10.1080/21541248.2017.1371268 (PMC6548291; doi:10.1080/21541248.2017.1371268)
Supplement: KSGT_A_1371268_supplemental.zip [file ksgt-10-03-1371268-s001.zip › KSGT_A_1371268_supplemental.pdf]

Recognition and stabilization of geranylgeranylated human Rab5 by the GDP Dissociation Inhibitor (GDI)

Eileen Edler and Matthias Stein

Supplementary Material

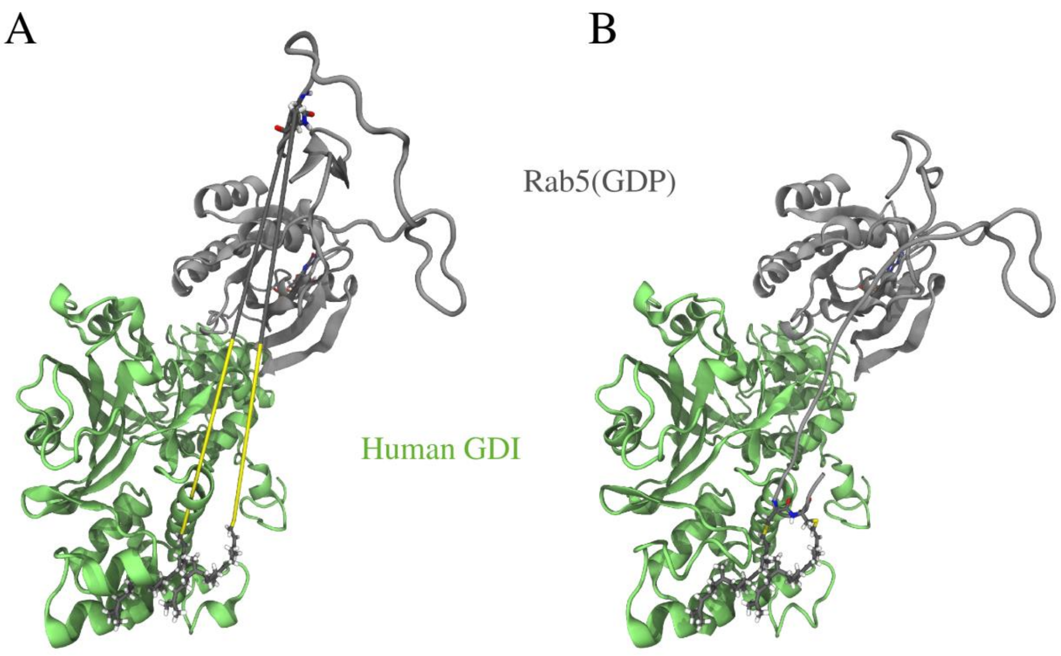

Fig.S1 Human Rab5(GDP):GDI complex before **(A)** and after energy minimization **(B).** The prenyl chain coordinates were constrained. Steric clashes are removed by minimization. Rab5(GDP) is coloured in grey, GDI is shown in green.

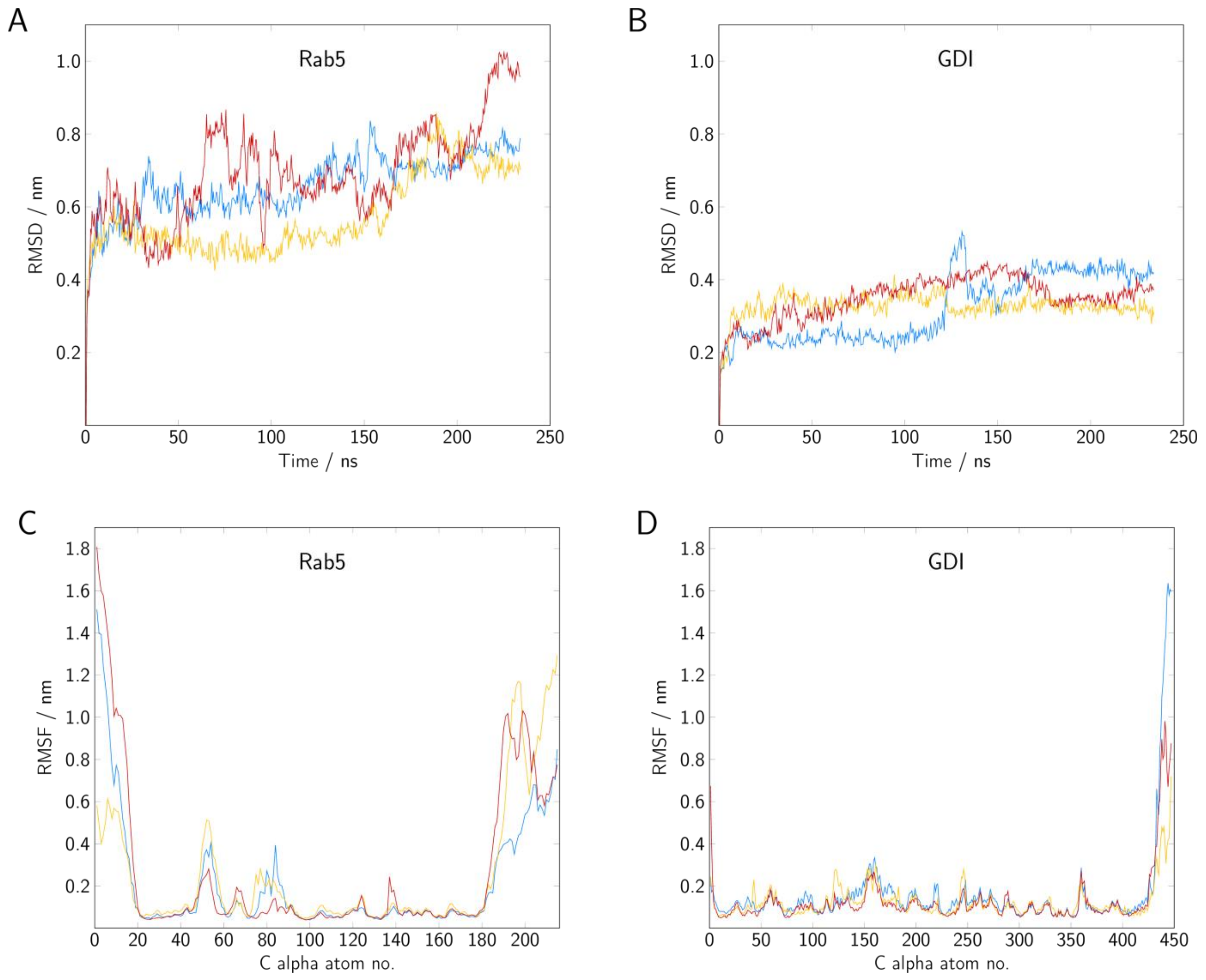

Fig.S2 The root mean square deviations (RMSD) of the cytoplasmic Rab5 G domain **(A)** and GDI **(B)** Cα atoms are shown over the full MD trajectory. The root mean square fluctuations (RMSF) of Rab5 **(C)** and GDI **(D)** Cα atom are calculated after superposition with the first frame coordinates. Results from the three individual runs are coloured in blue ($_{cyt}$Run1), yellow ($_{cyt}$Run2), and red ($_{cyt}$Run3).

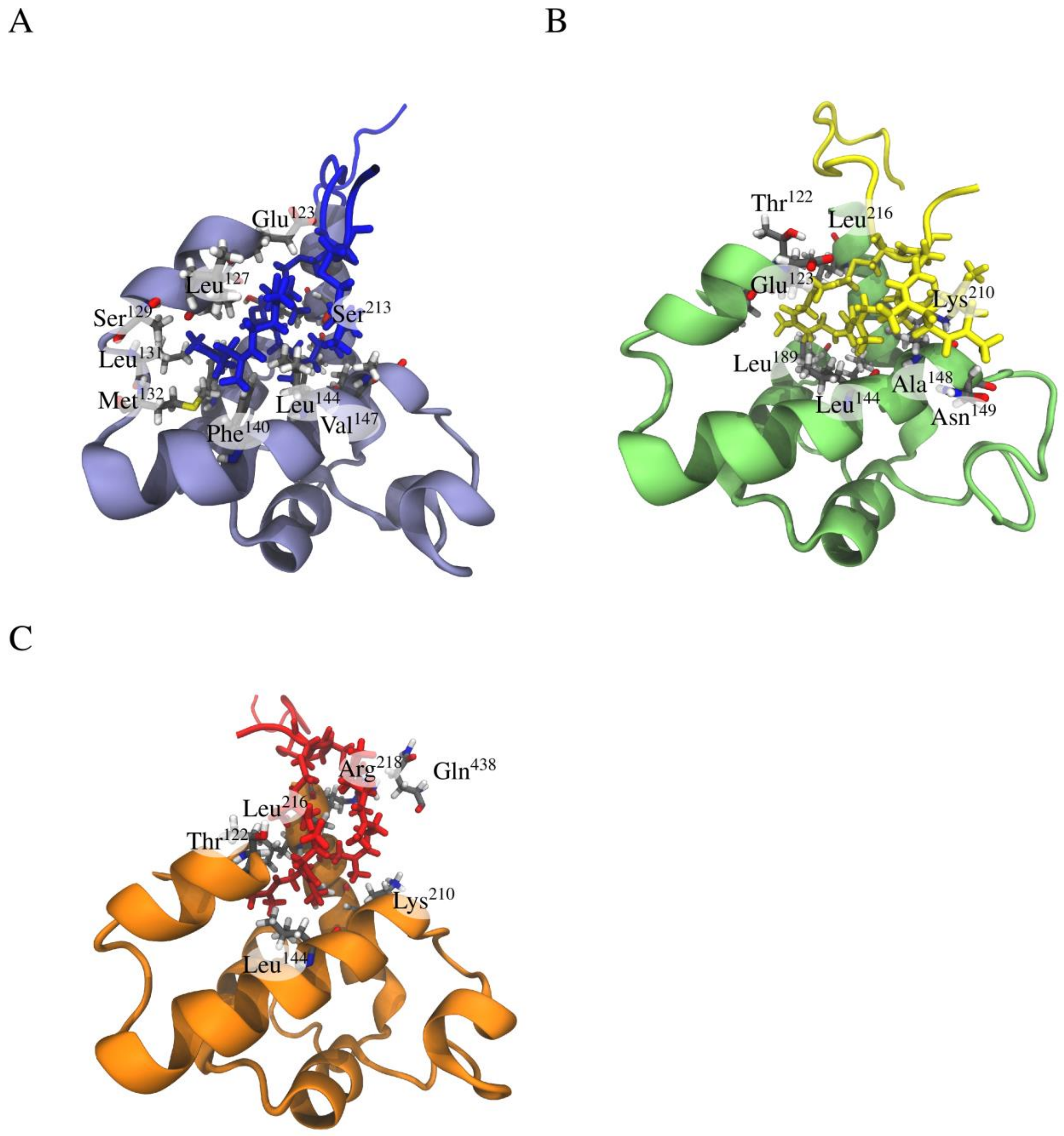

Fig.S3 (A-C) Representation of the interactions between specific GDI GG-binding pocket residues and the Rab5 GG chains after 250 ns of MD simulation. Results from $_{cyt}$Run1 are shown in light / dark blue, from $_{cyt}$Run2 in green / yellow, and from $_{cyt}$Run3 in orange / red.

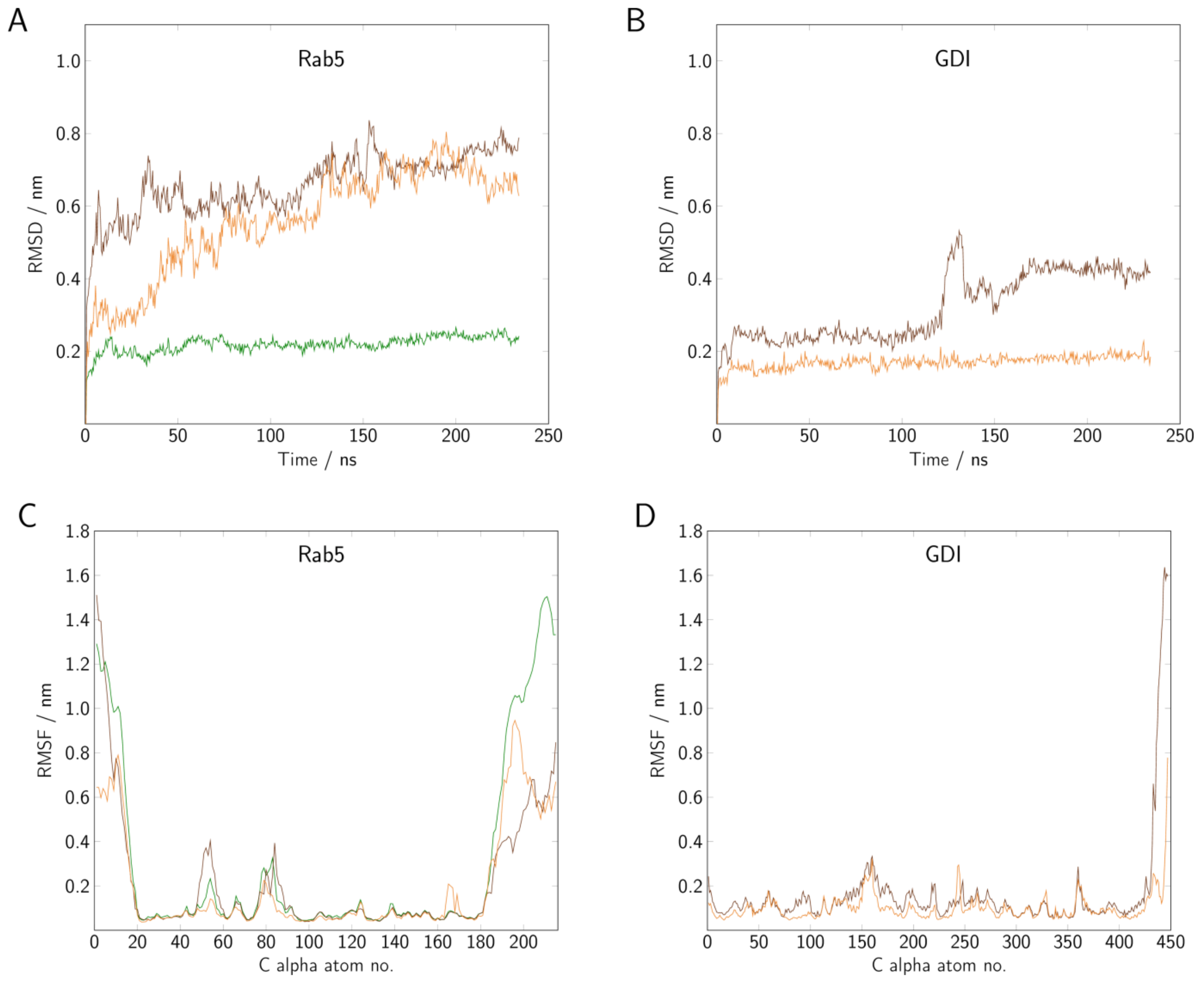

Fig.S4 Root mean square deviations (RMSD) of the Rab5 G domain **(A)** and associated GDI **(B)**. The Rab5 **(C)** and GDI **(D)** root mean square fluctuations (RMSF) were calculated over the entire trajectory. Colour coding is as follows: The green line corresponds to MD trajectories of free un-complexed membrane-bound Rab5(GDP) averaged over three previous simulations, the brown line represents the results of cytoplasmic Rab5(GDP) in complex with GDI in the tightly bound state ($_{cyt}$Run1) and the orange line shows the results with membrane-bound Rab5(GDP) in complex with GDI ($_{memb}$Run).

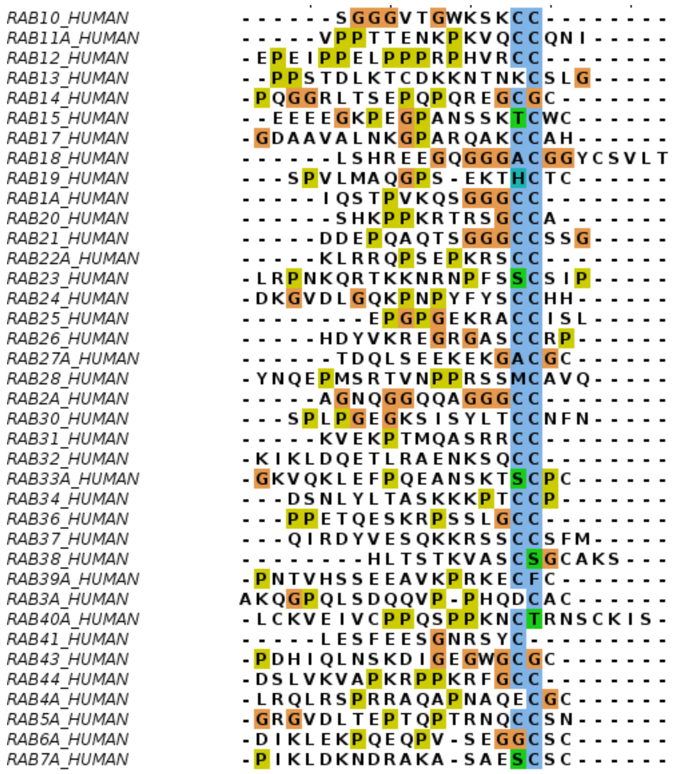

Fig.S5 Multiple sequence alignment of the C-terminal residues of the hypervariable region (HVR) of human Rab proteins.
